# Effects of Prolonged GRP78 Haploinsufficiency on Organ Homeostasis, Behavior, Cancer and Chemotoxic Resistance in Aged Mice

**DOI:** 10.1038/srep40919

**Published:** 2017-02-01

**Authors:** Amy S. Lee, Sebastian Brandhorst, Daisy F. Rangel, Gerardo Navarrete, Pinchas Cohen, Valter D. Longo, Jeannie Chen, Susan Groshen, Todd E. Morgan, Louis Dubeau

**Affiliations:** 1 Department of Biochemistry and Molecular Biology, University of Southern California Keck School of Medicine, USC Norris Comprehensive Cancer Center, 1441 Eastlake Avenue, Los Angeles, CA 90089-9176, United States; 2 Longevity Institute, Leonard Davis School of Gerontology and Department of Biological Sciences, University of Southern California, 3715 McClintock Avenue, Los Angeles, CA 90089-0191, United States; 3 Zilkha Neurogenetic Institute, Department of Cell and Neurobiology & Department of Ophthalmology, University of Southern California Keck School of Medicine, 1501 San Pablo Street, Los Angeles, CA 90033, United States; 4 Department of Preventive Medicine, University of Southern California Keck School of Medicine, 1441 Eastlake Avenue, Los Angeles, CA 90033, United States; 5 Department of Pathology, University of Southern California Keck School of Medicine, USC Norris Comprehensive Cancer Center, 1441 Eastlake Avenue, Los Angeles, CA 90089-9176, United States

## Abstract

GRP78, a multifunctional protein with potent cytoprotective properties, is an emerging therapeutic target to combat cancer development, progression and drug resistance. The biological consequences of prolonged reduction in expression of this essential chaperone which so far has been studied primarily in young mice, was investigated in older mice, as older individuals are likely to be important recipients of anti-GRP78 therapy. We followed cohorts of Grp78^+/+^ and Grp78^+/−^ male and female mice up to 2 years of age in three different genetic backgrounds and characterized them with respect to body weight, organ integrity, behavioral and memory performance, cancer, inflammation and chemotoxic response. Our results reveal that body weight, organ development and integrity were not impaired in aged Grp78^+/−^ mice. No significant effect on cancer incidence and inflammation was observed in aging mice. Interestingly, our studies detected some subtle differential trends between the WT and Grp78^+/−^ mice in some test parameters dependent on gender and genetic background. Our studies provide the first evidence that GRP78 haploinsufficiency for up to 2 years of age has no major deleterious effect in rodents of different genetic background, supporting the merit of anti-GRP78 drugs in treatment of cancer and other diseases affecting the elderly.

The 78-kilodalton glucose regulated protein 78 (GRP78), also referred to as BiP or HSPA5, is a stress-inducible molecular chaperone belonging to the heat shock protein (HSP) family^1^,^2^,^3^. While GRP78 shares 50% sequence homology with HSP70, which resides primarily in the cytosol, GRP78 contains a signal peptide that targets it to the endoplasmic reticulum (ER). GRP78 is the most abundant ER chaperone and serves critical functions in the translocation, folding and maturation of membrane and secretory proteins, as well as in the prevention of aggregation of unfolded and misfolded proteins and in targeting them for degradation^4^. GRP78 is also an ER calcium binding protein^5^. GRP78 is well established as a potent pro-survival factor under ER and proteotoxic stress conditions by sustaining ER protein folding capacity and maintaining ER stress sensors and ER-associated pro-apoptotic machineries in their inactive state^6^,^7^,^8^,^9^. GRP78 maintains ER integrity and suppresses stress-induced autophagy^10^. Furthermore, GRP78 can relocalize to the cell surface, the mitochondria and the nucleus, and even be secreted in a context dependent manner^11^,^12^,^13^,^14^. In these new cellular locations, GRP78 assumes new functions controlling cell-signaling, proliferation, survival, apoptosis, inflammation and immunity^5^,^11^. Thus, GRP78 integrity could be very important in maintaining normal cellular physiology and organ homeostasis during the life span of an organism.

GRP78 overexpression is widely reported in cancer cell lines and human cancer and is associated with aggressive growth and invasive properties^5^,^15^. Recently it was discovered that GRP78 modulates lipid metabolism to control drug sensitivity and antitumor immunity in cancer^16^. Furthermore, the upregulation of GRP78 in both tumor cells and the tumor-supporting stromal cells, as an adaptive response to stress, could represent a major obstacle to therapeutic efficacy^4^,^5^,^17^,^18^,^19^. Thus, GRP78 is extensively documented to confer resistance to chemotoxic drugs, anti-hormonal agents, DNA-damaging agents, anti-angiogenesis drugs and chromatin modifying drugs, as well as radiation therapy^5^,^15^,^20^,^21^,^22^,^23^. The protective effects of GRP78 have been observed in proliferating and dormant cancer cells, tumor initiating cells as well as tumor associated endothelial cells, including effects mediated not only by the ER form, but also the stress-induced cytosolic isoform, the secreted form and the cell surface form known to regulate the PI3K/AKT oncogenic signaling pathway^5^,^24^. Collectively, these findings indicate that targeting GRP78 in cancer could be a promising novel approach to combat this disease and prevent its resistance to therapy. Indeed, agents active against GRP78 in cancer, targeting both the intracellular and cell surface forms, have been developed and are being used in Phase I/II clinical trials^5^,^25^,^26^,^27^. Despite these exciting developments, the challenge is whether a therapeutic window can be established to minimize organ toxicity while maintaining its efficacy on tumor suppression. It is especially important to rule out organ toxicity in older individuals, who are the most likely recipients of such therapy.

We previously created conventional and conditional knockout mouse models of GRP78 and studied the biological consequences of whole body as well as tissue-specific GRP78 deficiency. We discovered that while complete absence of GRP78 is embryonic lethal and has major detrimental effects on organ function, embryos as well as adult mice bearing a single allele of GRP78 and thus expressing 50% of the wild-type level of this protein were phenotypically normal^3^,^28^. In contrast, tumor initiation, progression and neovascularization, as well as AKT signaling were substantially blunted in mice bearing GRP78 haploinsufficiency^5^,^29^,^30^,^31^. This implies that therapeutic agents suppressing GRP78 expression in the range of 30 to 50% might specifically impede tumor growth with minimal harmful effects on normal organs. Nonetheless, these studies were all performed in young adult mice.

It has been reported that aging leads to a significant decline in protein expression and activity of several ER chaperones, including GRP78^2^. For example, a reduction in GRP78 expression was observed in both brain and hepatic tissues of old versus young rodents, and is thought to contribute to age-related impairments in cellular function and pathologies^32^,^33^,^34^,^35^,^36^. We therefore examined aged WT and heterozygous Grp78 (Grp78^+/−^) male and female mouse cohorts in three different genetic backgrounds (C57BL/6, 129sv and mixed C57BL/6*129sv) to investigate the biological consequences and clinical implications of long-term GRP78 haploinsufficiency in aging individuals.

## Results

### GRP78 haploinsufficiency is sustained in organs throughout the life span of the Grp78^+/−^ mice

**Figure 1:**
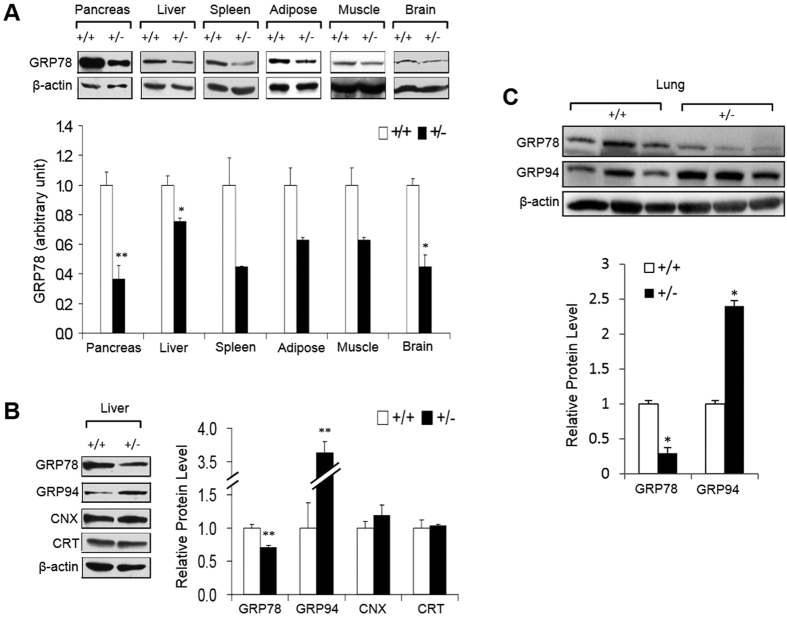
Sustained reduction of GRP78 protein level in organs of aged Grp78^+^/^−^ mice. (**A**) GRP78 protein levels in pancreas, liver, spleen, white adipose tissue, skeletal muscle and brain were examined by Western blotting in 7 month old Grp78^+/+^ and ^+/−^ mice of C57BL/6*129sv genetic background (n ≥ 3 for each genotype). Top panel: representative blots. Bottom panel: levels normalized against α-actin (muscle) or β-actin (other tissues) indicated as mean ± SEM. *unadjusted P < 0.05, **unadjusted P < 0.01. (**B**) Protein levels of ER chaperones GRP78, GRP94, calnexin (CNX), and calreticulin (CRT) were examined by Western blotting in liver of 7 month old Grp78^+/+^ and ^+/−^ mice of C57BL/6*129sv genetic background (n ≥ 3 for each genotype). Left panel: representative blots. Right panel: levels normalized against β-actin indicated as mean ± SEM. **unadjusted P < 0.01. (**C**) Protein levels of ER chaperones GRP78 and GRP94 from lung of 22 month old Grp78^+/+^ and ^+/−^ mice of C57BL/6 genetic background (n = 3 for each genotype). Top panel: representative blots. Bottom panel: levels averaged for each genotype and normalized against β-actin plotted as mean ± SEM. *unadjusted P < 0.05.

We previously reported that GRP78 protein level was reduced by half with compensatory upregulation of other ER chaperones such as GRP94 in Grp78^+/−^ mouse embryos and in livers of 6 week old mice with mixed C57BL/6*129sv genetic background^28^. At 7 months, mice of the same genetic background also showed about 50% reduction in GRP78 protein level in the majority of tissues, including the pancreas, spleen, adipose, muscle and brain, with slightly less reduction in the liver (Fig. 1A). A compensatory upregulation of GRP94 by about 3.5-fold was observed in the liver of Grp78^+/−^ mice, whereas the level of calnexin (CNX) and calreticulin (CRT) remained the same as in liver from the wild-type (Grp78^+/+^) mice (Fig. 1B). In 2-year-old Grp78^+/−^ mice, backcrossed to a pure C57BL/6 genetic background, the level of GRP78 in the lung and other organs was also reduced by about 50% with upregulation of GRP94 by about 2.5-fold (Fig. 1C and data not shown). We conclude that in the Grp78^+/−^ mouse model, GRP78 haploinsufficiency is sustained throughout the life span in two different genetic backgrounds, making these models suitable to analyze the consequences of chronic partial depletion of GRP78 in aged mice.

### Aged Grp78^+/−^ mice showed no alterations in body weight, organ size and morphology

**Figure 2:**
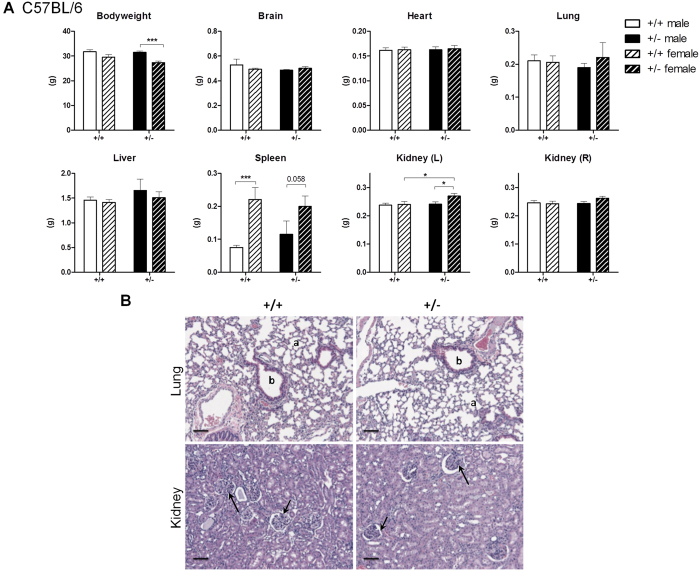
Aged C57BL/6 Grp78^+^/^−^ mice exhibited normal body weight, organ size and morphology. (**A**) 23–25 month old C57BL/6 mice were sacrificed and body/organ weights were measured and plotted as mean ± SEM. Male: Grp78^+/+^ (n = 13); Grp78^+/−^ (n = 18); female: Grp78^+/+^ (n = 11); Grp78^+/−^ (n = 11). *unadjusted P < 0.05, ***unadjusted P < 0.001. (**B**). Representative histological sections of lung and renal cortex of the indicated genotypes. Both organs show normal tissue architecture. Lungs of GRP78^+/+^ and GRP78^+/−^ mice show unremarkable alveolar spaces (a) of normal sizes and lined by alveolar septae of similar and normal thickness. Bronchioles (b) are lined by respiratory epithelium. The kidneys show glomeruli (arrows) with normal cellularity and unremarkable renal tubules. Magnification bars: 100 microns.

In the C57BL/6 background, no substantial differences in total body weight or in the weight of individual organs including brain, heart, lung or liver were noted between 2-year-old WT and Grp78^+/−^ mice (Fig. 2A). Nonetheless, female Grp78^+/−^ mice tended to be smaller than males of the same genotype (*p* < 0.001 – significant after using the Benjamini-Hochberg false discovery rate method with α = 0.05). The spleen of female Grp78^+/−^ mice weighed almost twice as much as that of male Grp78^+/−^ mice (*p* = 0.058), however, this was not related to GRP78 expression because the same trend was observed in WT male and female mice (*p* = 0.0024 – significant after using the Benjamini-Hochberg false discovery rate). There was a significant increase in the weight of the left kidney, but not of the right kidney in female Grp78^+/−^ mice compared to the female WT mice (Fig. 2A). No histological change was noted that could account for these differences in renal weight. Sections of lung and kidney from WT and Grp78^+/−^ mice showed normal architecture in both genotypes (Fig. 2B), including the number and cellularity of renal glomeruli. The lungs of Grp78^+/−^ mice showed no appreciable fibrosis and the thickness of alveolar septae were similar thickness to those of WT mice. Thus, there was no morphological evidence of any difference in pulmonary or renal architecture between aged C57BL/6 WT and Grp78^+/−^ mice.

**Figure 3:**
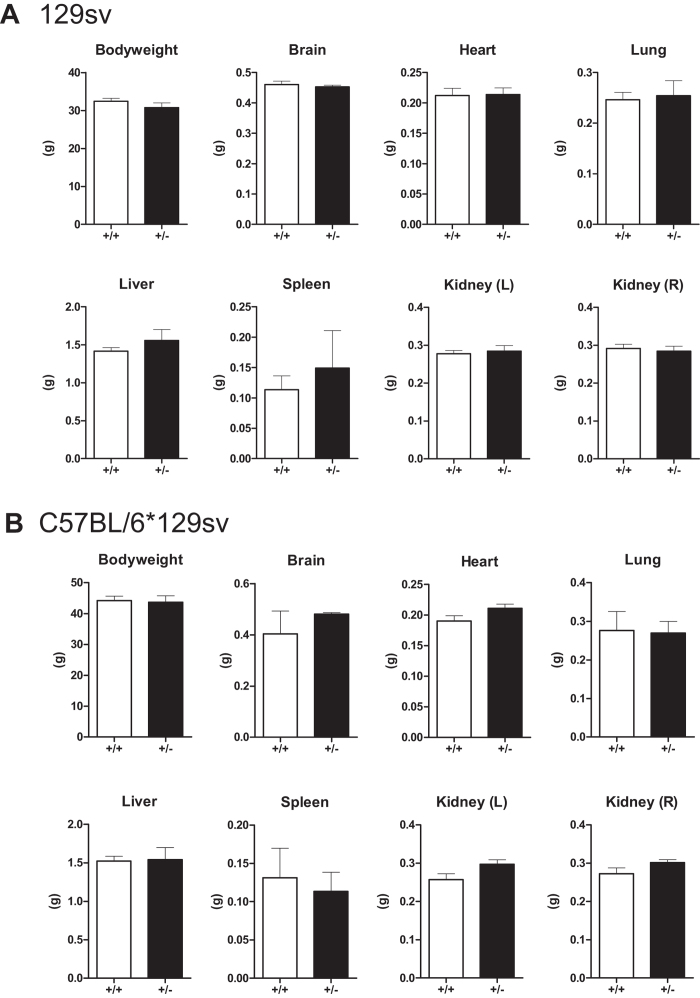
Similar body weight and organ size in aged Grp78^+^/^−^ mice of 129sv and C57BL/6*129sv genetic backgrounds. 23–25 month old male (**A**) 129sv mice or (**B**) C57BL/6*129sv mice were sacrificed and body/organ weights were measured and plotted as mean ± SEM. (**A**) Grp78^+/+^ (n = 3); Grp78^+/−^ (n = 9). (**B**) Grp78^+/+^ (n = 5); Grp78^+/−^ (n = 6).

We expanded these analyses to mice in other genetic backgrounds and detected no significant changes in total body weight or organ weight of brain, heart, lung, liver, spleen and kidneys of male mice in the pure 129sv background (Fig. 3A) or mixed C57BL/6*129sv background (Fig. 3B). We conclude that chronic GRP78 haploinsufficiency maintained over two years had no deleterious effects on growth and organ development in mice of different genetic backgrounds.

### Lack of deleterious effects of prolonged GRP78 haploinsufficiency on circulating levels of glucose and IGF-I in aged mice

**Figure 4:**
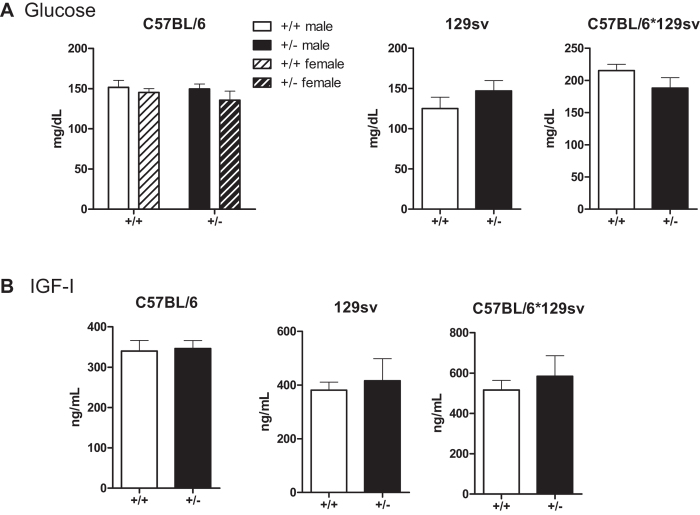
Normal circulating levels of glucose and IGF-I in aged Grp78^+^/^−^ mice. (**A**) Serum glucose levels in 23-25 month old mice of the indicated genotypes, genders, and genetic backgrounds. C57BL/6 [male: Grp78^+/+^ (n = 13); Grp78^+/−^ (n = 18); female: Grp78^+/+^ (n = 11)]; Grp78^+/−^ (n = 11). (**B**) 129sv: [male: Grp78^+/+^ (n = 3); Grp78^+/−^ (n = 9); female: Grp78^+/+^ (n = 7)] or (**C**) C57BL/6*129sv mice [male: Grp78^+/+^ (n = 5); Grp78^+/−^ (n = 6)].

We measured the serum levels of glucose and IGF-I in the aged mouse cohorts following 4 h of fasting to avoid changes related to food consumption. Two-yr-old Grp78^+/−^ female and male C57BL/6 mice displayed no significant difference in blood glucose levels compared to the same genders in WT mice. Similar observations were made with 129sv and mixed C57BL/6*129sv male mice (Fig. 4A). In all three genetic backgrounds (C57BL/6, 129sv and mixed C57BL/6*129sv) tested, no difference in IGF-I level was detected. Thus, GRP78 haploinsufficiency over a period of up to 2 years had no impact on circulating glucose and IGF-I levels (Fig. 4B).

### Lack of deleterious effects of prolonged GRP78 haploinsufficiency on circulating levels of hematopoietic cells in aged mice

**Figure 5:**
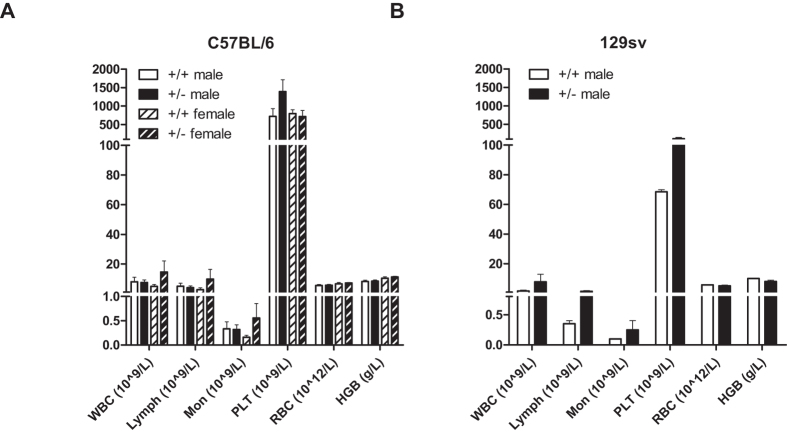
Number of circulating hematopoietic cells in aged Grp78^+^/^−^ mice. Complete blood cell counts were obtained for 23–25 month old (**A**) C57BL/6 mice and (**B**) 129sv mice. White blood cells (WBC), lymphocytes (Lymph), monocytes (Mon), platelets (PLT), red blood cells (RBC) and hemoglobin (HGB) levels are shown. (**A**) Male Grp78^+/+^ (n = 9), male Grp78^+/−^ (n = 10), female Grp78^+/+^ (n = 5) and female Grp78^+/−^ (n = 7). (**B**) Male Grp78^+/+^ (n = 2), and Grp78^+/−^ (n = 6). Bars: mean ± SEM.

Complete blood cell counts were performed on 2-year-old C57BL/6 male and female mice and on 129sv male mice; in general, the 18 blood profile parameters examined were highly similar across the different groups (Fig. 5 and Supplemental Table S1). Minor differences included: the C57BL/6 female Grp78^+/−^ mice showed mildly elevated levels of total white blood cells, primarily due to slight increase in lymphocyte and monocyte levels, whereas platelet levels were increased in Grp78^+/−^ male mice (Fig. 5A). Similarly, in the 129sv background platelet levels were increased in male Grp78^+/−^ mice (Fig. 5B). Total white blood cells were also slightly increased in heterozygote 129sv males, primarily due to increase in lymphocyte and monocyte levels. None of these differences were statistically significant at 0.05-level except for the increased platelet counts in both the C57BL/6 and 129sv Grp78^+/−^ male mice, which remained statistically significant after adjusting with Tukey’s HSD (*p* < 0.05). Thus, only subtle differences were observed between the two genotypes, dependent on gender and genetic background.

### Retinal structure and function is intact in aged Grp78^+/−^ mice

**Figure 6:**
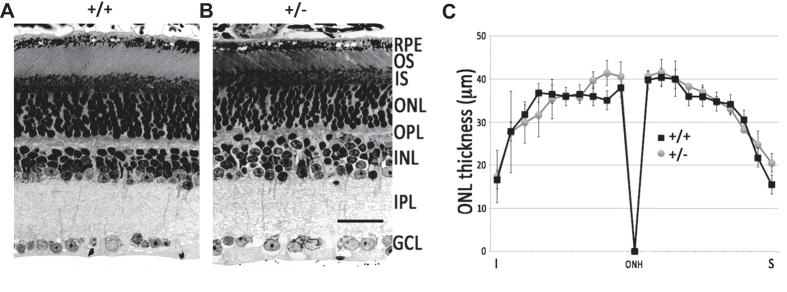
Normal retinal morphology in 2-year-old Grp78^+^/^−^ mice. Light micrograph of retinal sections from 2-year-old C57BL/6 Grp78^+/+^ mice (**A**) and Grp78^+/−^ mice (**B**). RPE, retinal pigmented epithelium; OS and IS, photoreceptor outer segment and inner segment; ONL, outer nuclear layer; OPL, outer plexiform layer; INL, inner nuclear layer; IPL, inner plexiform layer; GCL, ganglion cell layer. Scale bar = 40 μm. (**C**) Outer nuclear layer thickness was measured at 20 different positions along the inferior (I) and superior (S) axis along the central meridian for control (Grp78^+/+^, N = 3) and Grp78^+/−^ (N = 4) mice. The plot shows average thickness ± SD.

Although ER stress and reduced GRP78 levels have been implicated in many retinal diseases, and GRP78 augmentation by viral delivery can reverse these pathologies^37^, the effect of long term GRP78 haploinsufficiency on retinal physiology in aged rodents is not known. Retinal sections were prepared from 2-year-old C57BL/6 WT and Grp78^+/−^ mice. No differences in morphology were observed in any retinal layers (Fig. 6A and B). Because altered GRP78 levels have been observed in different forms of retinal degenerations associated with photoreceptor cell death^38^,^39^, we measured the thickness of photoreceptor nuclei, outer nuclear layer (ONL) along the inferior to superior axis along the vertical meridian to detect differences across the entire span of the retina. No difference in ONL thickness was detected between WT and Grp78^+/−^ mice (Fig. 6C). To assess retinal function, WT and Grp78^+/−^ mice were subjected to electroretinography, where light-induced a-wave reflects summed responses from photoreceptors and b-wave reflects the activity of downstream bipolar cells. No differences in the electroretinogram were observed between WT and Grp78^+/−^ mice (data not shown).

### GRP78 haploinsufficiency has no major negative outcome on behavioral performance

**Figure 7:**
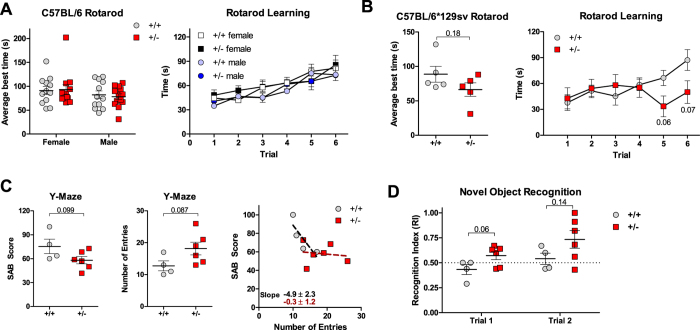
Behavioral performance of aged Grp78^+^/^−^ mice. **(A)** Best Rotarod performance score in 19–22 month old female and male C57BL/6 Grp78^+/+^ and Grp78^+/−^ mice. N = 12-13/group. Rotarod learning performance over the 6 trial period is shown for each gender and genotype. (**B**) Best Rotarod performance score in 23 month old male C57BL/6*129sv Grp78^+/+^ and Grp78^+/−^ mice. N = 5/group. Rotarod learning performance over the 6 trial period is shown for each genotype. (**C**) Spontaneous alternation behavior (SAB) and number of arm entries in the Y-maze for C57BL/6*129sv Grp78^+/+^ and Grp78^+/−^ male mice. N = 4-6/group. (**D**) Recognition index at 23 month for C57BL/6*129sv Grp78^+/+^ and Grp78^+/−^ male mice in the novel object recognition task. Exploration time of the old vs. old (trial 1) or novel object (trial 2). N = 4-6/group. Each data point represents mean ± SEM.

We next tested whether GRP78 haploinsufficiency might have an impact on age-related deficits in motor function, learning and memory. In the C57BL/6 background, we compared motor coordination in 19–22 month old female and male WT and Grp78^+/−^ mice using the Rotarod test. Performance was measured for two variables: average of the best daily performance in two consecutive trial days, and average of individual times a mouse remained in balance over six trial sessions. There were no significant differences in the best time or training index between gender and genotype for C57BL/6 mice (Fig. 7A). Similarly, female C57BL/6*129sv WT and Grp78^+/−^ mice showed no significant difference in overall best time performance (*p* = 0.18; Fig. 7B) although Grp78^+/−^ mice displayed training difficulties during the last trials (trial 5 *p* = 0.06, trial 6 *p* = 0.07).

C57BL/6*129sv males (23 months old) were evaluated using the Y-maze test and the Novel Object Recognition test. Using the Y-maze test, the Spontaneous Alternation Behavior (SAB) score was calculated as the proportion of alternations (an arm entry differing from the previous two entries) in the total number of alternation opportunities. There was a trend suggesting that WT mice had higher SAB scores (*p* = 0.1) but lower numbers of arm entries (a measure of overall activity, *p* = 0.09), compared to the Grp78^+/−^ mice (Fig. 7C). When comparing the SAB score to the number of arm entries, we noted that WT mice reduced their score with an increased number of arm entries. Notably, Grp78^+/−^ mice did not display this behavior but had lower SAB scores throughout the trial (Fig. 7C). No differences in novel object recognition (calculated as time in seconds) spent between familiar and new objects were detected (Fig. 7D). Collectively, the above tests indicate that GRP78 haploinsufficiency has generally neutral outcomes on a variety of behavioral tasks, although there might be some minor negative strain-specific effects for selected learning tasks.

### Lack of consequences of prolonged Grp78 haploinsufficiency on incidence of cancer and inflammation in aged mice

**Figure 8:**
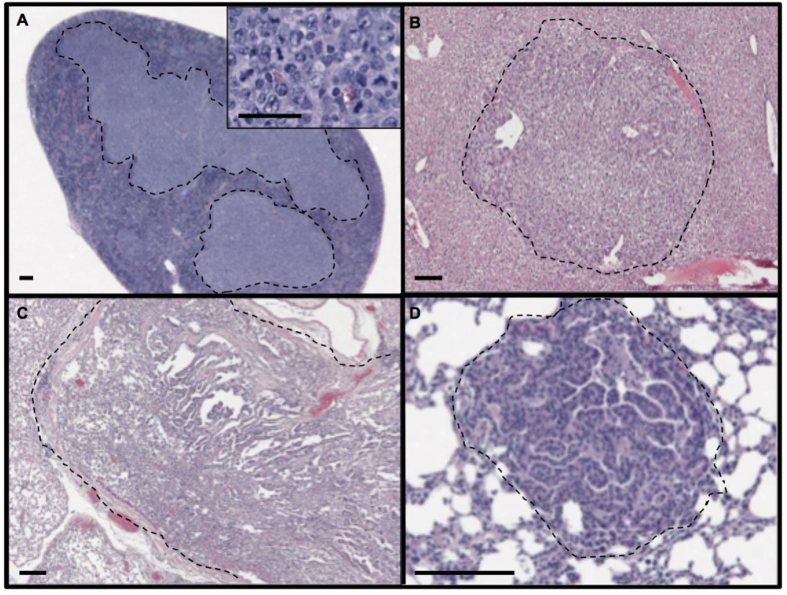
Representative examples of neoplastic tumors in aged Grp78^+^/^+^ and Grp78+/− mice. (**A**) Section of spleen from a Grp78^+/+^ mouse showing extensive infiltration by a lymphoma outlined by the dashed lines; the inset shows a magnified representative section of the tumor, illustrating a monoclonal population of cells with pleomorphic nuclei and a high mitotic rate. (**B**) Section of liver from a Grp78^+/−^ mouse showing an adenoma outlined by the dashed line. Sections of lung from Grp78^+/+^ (**C**) and Grp78^+/−^ (**D**) mice. In each case the pulmonary parenchyma is infiltrated by an adenocarcinoma, which is extensive in (**C**) and microscopic in (**D**). Both tumors show papillary architecture. Tumors in panels **A** and **D** are from C57BL/6 mice while those in panels **B** and **C** are from C57BL/6*129sv mice. Magnification bars: 50 microns in **A**, 100 microns in (**B–D**).

Aged mice of the two genotypes were necropsied immediately after euthanasia. All organs were examined macroscopically and those with gross abnormalities were submitted for histological examination. In addition, the following organs were submitted for such examination regardless of macroscopic findings: liver, lung, kidney, pancreas, heart and brain. Liver adenomas and adenocarcinomas, which are considered part of the same disease continuum, were scored as a single entity with no further attempt to distinguish them based on malignant potential. Tumors from other organs were all invasive, malignant lesions. Representative examples of neoplastic tumors in WT and Grp78^+/−^ mice are shown in Fig. 8. In the pure C57BL/6 (n = 32) and mixed C57BL/6*129sv (n = 11) strains, twice as many WT mice developed tumors than Grp78^+/−^ mice; however, this pattern was not statistically significant. Although a larger proportion of Grp78^+/−^ 129sv mice developed tumors than their wild-type counterpart, these differences were also not statistically significant (Table 1).

In all three strains, several mice had developed some form of inflammation in at least one organ at the time of death, either chronic (most common) or acute (Table 2). In the pure 129sv (*n* = 14) and mixed C57BL/6*129sv (*n* = 11) strains, similar proportions of WT and Grp78^+/−^ mice showed inflammation in specific organs while in the pure C57BL/6 strain (*n* = 32) a higher proportion of Grp78^+/−^ mice (85%) developed inflammation compared to WT mice (47%); none of the comparisons between WT and Grp78^+/−^ mice were statistically significant after controlling for a false discovery rate of 0.05. Renal inflammation was seen in 4 of 13 Grp78^+/−^ in the C57BL/6 background compared to none of the 19 wild-type mice with the same genetic background. Although comparison of these two proportions (4/13 *vs.* 0/19) using Fisher’s exact test showed a P value of 0.020, this difference was not statistically significant after adjusting for a 0.05 false discovery rate.

Steatosis can reflect an underlying inflammatory condition of the liver or can be a general sign of hepatic failure. The presence of steatosis in the absence of a significant inflammatory infiltrate was not scored as inflammation in Table 2. This condition was seen predominantly in mice with mixed C57BL/6*129sv background and may therefore be strain-specific. It was not associated with Grp78 status (2 of 5 Grp78^+/+^ and 2 of 6 Grp78^+/−^). The only other example of steatosis without inflammation seen in our entire mouse population was in a single wild-type mouse of pure 129sv background. Thus, prolonged Grp78 haploinsufficiency does not result in liver damage associated with steatosis.

### Chemotoxic stress response in aged male and female Grp78^+/−^ mice

**Figure 9:**
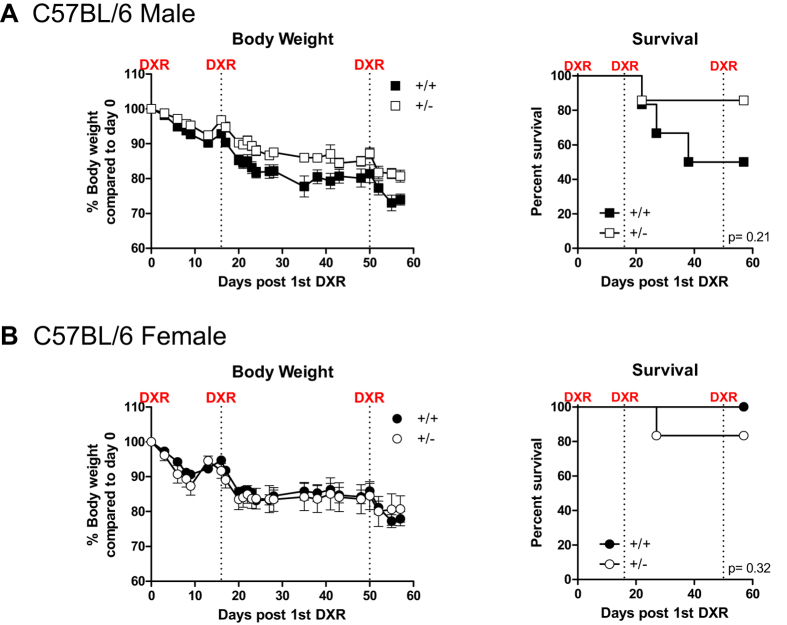
Chemotoxic stress response of aged Grp78^+^/^−^ mice. (**A**) male and (**B**) female 20–22 month old C57BL/6 Grp78^+/+^ and Grp78^+/−^ mice received 8 mg/kg intravenous inoculations of Doxorubicin (DXR) at the time points indicated by dashed lines. Chemotoxicity-induced weight loss and survival are presented for each gender and genotype. N = 6-7/group.

We next tested the effect of Grp78 haploinsufficiency on chemotoxic stress response in 20 to 22 month old male and female C57BL/6 mice. Three 8 mg/kg injections of the chemotherapeutic drug doxorubicin were given on days 1, 15 and 50 (Fig. 9). Toxicity-induced weight loss was more pronounced in male WT mice, who also showed 50% survival 60 days after the first doxorubicin injection compared to the 85% in the Grp78^+/−^ cohort (*p* = 0.21, Fig. 9A). For female WT and Grp78^+/−^ mice, chemotoxicity-induced weight loss and survival were comparable between 80 and 100% at 60 days following the first doxorubicin injection (Fig. 9B). This apparent trend in gender-specific differences did not reach statistical significance.

## Discussion

Given its potent cytoprotective properties, GRP78 is an emerging target for therapy to blunt cancer development, progression and drug resistance^5^,^16^,^27^. We sought to characterize the effects of chronic partial GRP78 depletion in aged mice, as age is an independent risk factor for cancer and other human diseases and given that previous studies on the role of GRP78 in organ function and cancer were focused mostly on young mice. In addition, the issue of whether the genetic background plays any role in the requirement of GRP78 for maintenance of organ homeostasis and protection has not been adequately addressed. We established cohorts of aged WT and Grp78^+/−^ male and female mice of three different genetic backgrounds and characterized them with respect to body weight, organ integrity, behavioral performance, cancer, inflammation and chemotoxic response. Our goal was to determine whether a prolonged 50% reduction in GRP78 over their life span would be detrimental, inconsequential or protective regarding selected parameters in aged mice. Our results reveal that in general, body weight and organ development and integrity were not impaired in aged GRP78^+/−^ mice. No statistically significant effect on spontaneous cancer incidence and inflammation was observed. This is the first evidence that GRP78 haploinsufficiency over a period of up to two years has no major deleterious effect in aged rodents of different genetic backgrounds.

Subtle differential trends between the WT and Grp78^+/−^ mice were observed in some test parameters dependent on gender and genetic background, reflecting heterogeneity in complex health and disease indexes in aging populations. For example, in both C57BL/6 and 129sv male mice, Grp78 heterozygosity resulted in higher numbers of circulating platelets. While the cause could be complex and multifactorial, high platelet counts could occur due to an underlying infection or cancer. Indeed, Grp78^+/−^ mice in the C56BL/6 and 129sv background showed a trend toward higher incidences of inflammation and cancer compared to the WT mice. In behavioral performance tests, we noted that female C57BL/6*129sv Grp78^+/−^ mice displayed training difficulties during the last trials and that male mice of the same genotype showed lower spontaneous alternation behavior scores throughout the trials compared to WT mice. This suggests that GRP78 haploinsufficiency could have some minor negative effects on selected learning tasks. WT male mice had a worse chemotoxic response as evidenced by increased weight loss and decreased survival compared to the female WT mice. Higher sensitivity of male vs. female rodents to oxidative stress was commonly observed in experiments performed with young animals^40^. Similarly, in our aged cohorts, we observed a higher sensitivity of males to chemotoxic stress compared to females. We noted that while aged Grp78^+/−^ females showed comparable chemotoxic response to the WT mice, aged Grp78^+/−^ male mice showed trends toward less severe weight loss and increased survival. The exact significance of this possible beneficial effect of partial GRP78 depletion awaits future studies with larger mouse cohorts.

The rod photoreceptor is a sensory neuron specialized in light detection. Each rod contains ~5 × 10^7^ rhodopsin molecules, of which 10% at the outer segment tip is phagocytosed daily by the RPE^41^,^42^. Therefore each rod must synthesize ~5 × 10^6^ rhodopsin molecules per day in the ER, together with other abundantly expressed phototransduction proteins. This unusually high demand of protein synthesis in the ER may place a high demand for chaperones such as GRP78 for normal retinal physiology. We observed that retinal morphology and function were not affected by GRP78 haploinsufficiency in aged mice, suggesting that retinal GRP78 levels are expressed in excess during normal aging. This conclusion may also apply to the other organs examined in this study.

There could be multiple reasons why loss of 50% of GRP78 in aged mouse tissues did not lead to any major detrimental outcome. In addition to only low basal levels of GRP78 being needed for normal organ function, we previously observed up-regulation of GRP94, another ER chaperone with key roles in receptor and growth factor maturation and secretion^43^, as an apparent compensatory response to the partial loss of GRP78 in mouse embryos^28^ and in pancreas of young mice^44^. This was confirmed in various organs of aged Grp78^+/−^ mice in this study. Thus, in a context dependent manner, GRP78 haploinsufficiency could lead to some negative outcome in the absence of a compensatory increase in GRP94 and other adaptive responses. On the other hand, adaptive responses such as upregulation of other ER chaperones stimulated by GRP78 decrease were shown to confer beneficial protective effect for cell metabolism in a tissue-specific manner^45^. Therefore, preventing a compensatory increase in GRP94 by targeting this protein in addition to GRP78 might be a more effective therapeutic approach in specific situations.

In summary, our findings that prolonged Grp78 haploinsufficiency had no major consequences in aged mice strongly suggests that targeting GRP78 for treatment of diseases like cancer is unlikely to have major deleterious side effects. Our observations were reproduced in mice of different genetic backgrounds and gender. Our findings are consistent with the notion that normal organ function requires only a low basal GRP78 level for maintenance, while cancer cells require high levels of GRP78 for growth, survival, invasion and therapeutic resistance. Small molecule and RNAi inhibitors capable of suppressing GRP78 expression or activity have recently been identified and reported to lower survival and alleviate drug and radio resistance in a wide variety of cancer cells^5^,^27^,^46^,^47^,^48^,^49^. Our results increase the merit of targeting GRP78 for the treatment of cancer and of other diseases dependent on high levels of GRP78.

## Methods

### Mouse strains

The construction of the Grp78 heterozygous (Grp78^+/−^) mice was described^28^. Male and female mice with C57BL/6, 129sv and the C57BL/6*129sv genetic backgrounds were used. All animal protocols were approved by the Institutional Animal Care and Use Committee (IACUC) of the University of Southern California. All experiments were performed in accordance with relevant guidelines and regulations. All mice were maintained in a pathogen-free environment and housed in clear shoebox-cages in groups of three animals per cage with constant temperature and humidity and 12 h/12 h light-dark cycle with unlimited access to water.

### Physiological biomarkers

Prior to blood collection and glucose measurements, mice were withheld from food for up to 4 h to avoid interferences caused by food consumption. Blood glucose was measured with the Precision Xtra blood glucose monitoring system (Abbott Laboratories). Mouse serum IGF-I was measured using a mouse specific ELISA kit (R&D Systems). Body weight of individual animals was measured regularly throughout the study and organ weight was recorded upon euthanasia.

### Complete blood count measurements

Complete blood cell counts were measured using a Mindray BC-2800 VET auto hematology analyzer following the manufacturer’s protocol. In brief, blood was collected from the tail vein in heparin-coated micro-hematocrit tubes. Twenty μl of the heparinized blood was added to CDS diluent (Clinical Diagnostics Solution Inc.) and whole blood parameters were evaluated.

### Western blot analysis

Lysates of tissues from individual mice were extracted by 3 freeze-thaw cycles followed by homogenization in ice-cold radioimmunoprecipitation assay buffer (50 mmol/l Tris-Cl, 150 mmol/l NaCl, 1% NP-40, 0.5% sodium deoxycholate, and 0.1% SDS) containing cocktails of proteinase inhibitors and phosphatase inhibitors (Roche), and centrifuged for 15 min at 13,000 *g*. Proteins were separated by 8% or 12% SDS-PAGE, transferred to nitrocellulose membrane (Pall), and subjected to Western blotting^10^. Primary antibodies included GRP78, GRP94, CNX and CRT (1:2,000; Stressgen), and β-actin (1:5,000; Sigma). Western blotting was repeated two to four times and signal intensities were measured using the Quantity One system (Bio-Rad).

### Histological examination

Mice were necropsied immediately after euthanasia. All organs were examined macroscopically and those with gross abnormalities were submitted for histological examination. In addition, the following organs were submitted for such examination regardless of the presence or absence of macroscopic findings: liver, lung, kidney, pancreas, heart and brain. Organs submitted for histological examination were fixed in 10% buffered formalin after being sliced into 1 mm sections to ensure good penetration by the fixative. The tissues were loaded on an automated tissue processor and incubated in increasing amounts of alcohol followed by impregnation with xylene. The tissues were then embedded in paraffin. Five micron-thick sections were obtained from the paraffin blocks using a microtome, stained with hematoxylin and eosin, and examined histologically by a Board Certified Pathologist familiar with mouse histopathology (LD). Tissues not submitted for histological examination were fixed for 48 h in 10% buffered formalin and stored in 70% ethanol.

### Retinal morphology

The superior pole of the cornea was marked by cauterization and enucleated eyes were placed in fixative (2.5% glutaraldehyde, 2.0% paraformaldehyde in 0.1 M cacodylate buffer, pH 7.2). The cornea and lens were removed and the remaining eyecup was processed into epoxy resin. The retina was sectioned along the superior-inferior axis. One micron-thick sections including the region of the optic nerve were stained, and outer nuclear layer thickness was measured as described^50^.

### Behavior tests

Three behavioral tests were used to compare WT to GRP^+/−^ mice^51^.

### Y-maze (Spontaneous Alternation Behavior)

Short-term spatial recognition memory was examined by a spatial novelty preference task in the Y-maze. The Y-maze was made of black plexiglass and comprised of three identical arms (50 × 9 × 10 cm), radiating from a central triangle (8 cm on each side) and spaced 120° apart from each other. The test started by placing the animal in one of the arms of the maze. The mouse was allowed to freely explore the environment for 8 min. The total number of arm entries (a measure of overall activity) was recorded for each mouse. An arm entry is defined as both fore-paws and hind-paws fully entering an arm of the Y-maze. The Spontaneous Alternation Behavior (SAB) score was calculated as the proportion of alternations (an arm entry differing from the previous two entries) in the total number of alternation opportunities. This test was performed once for each mouse (males: +/+ N = 4, +/− N = 6).

### Rotarod test

The Accelerating Rotarod was used to measure motor coordination. The device for this test consists of a rotating rod 3 cm in diameter suspended 15 cm above the base and divided by flanges so that up to 5 mice can be tested simultaneously. Mice were placed on the rotating rod and the speed gradually increased from 4 rpm to 40 rpm within a 5 min session; the rate of increase of speed was identical in all trials. The exact length of time during which the mice were able to stay on the bar was recorded. On two consecutive days, the mice were given three successive trials, for a total of six trials. Performance was measured for two variables: the average of the daily best time over the two trial days, and the individual lengths of time the mouse remained in balance for each of the six trials.

### Novel object recognition

The novel object recognition test was introduced to assess the ability of rodents to recognize a novel object in a familiar environment. The test includes a habituation phase (5 min on day one) and two trial phases (5 min each on the second day) for each mouse. In the habituation phase, the mouse was placed into a rectangular cage (50 × 50 × 40 cm) made of black acryl plexiglass for 5 min without any object. The testing session comprised two trials of 5 min each. Mice (males: +/+ N = 4, +/− N = 6) were always placed in the apparatus facing the wall in the middle of the front segment. In the first trial (T1), two identical objects were placed in the cage and the time spent exploring these objects was recorded. Exploration of the objects is defined as any physical contact with an object (whisking, sniffing, rearing on or touching the object) as well as positioning its nose toward the object at a distance of less than 2 cm; however, sitting or standing on top of the object was not recorded as exploration time. After the first exploration period (T1), the mice were placed back in their home cage. To control for odor cues, the open field arena and the objects were thoroughly cleaned with water, dried, and ventilated for a few minutes between tests. After a 1 h delay interval, mice were placed back in the apparatus for the second trial (T2), but now with two dissimilar objects, a familiar one and a new one. The time spent exploring both objects were recorded manually. The recognition index (RI) at each trial (T1 and T2) was calculated as the time (in seconds) spent exploring the familiar object divided by the total time spent exploring either one of the 2 objects; in T1, the object in the same location as the familiar object in T2 was designated as the familiar object.

### Chemotherapy treatment

C57BL/6 mice were intravenously injected via the lateral tail vein with Doxorubicin (DXR, Bedford Laboratories) and monitored daily. The mice (ages 20 to 22 mo) received 3 bolus injections of 8 mg/kg each on Days 1, 15 and Day 50. Animals showing signs of severe stress and/or deteriorating health status were designated as moribund and euthanized. Weights were recorded daily and the day of death (spontaneous or due to euthanasia) was recorded.

### Statistical methods

Unless otherwise specified, all results are expressed as the mean ± standard error of the mean. Differences among groups were tested either by Student’s two-sample *t*-test or the *F*-test based on a two-way analysis of variance (background-sex by genotype) followed by Tukey’s method for adjusting for multiple comparisons [honest significant difference (HSD)]. Kaplan-Meier survival curves were constructed to display survival for each group; groups were compared using the Wilcoxon test. When multiple endpoints or multiple subsets were examined for a particular analysis, to control for multiple testing, the Benjamini-Hochberg false discovery rate method was used, with a false discovery rate = 0.05^52^. All reported p-values are the nominal two-sided p-values; those that meet the Benjamini-Hochberg criteria and remain “significant” are so indicated in the tables and text. Nominal (unadjusted) p-values < 0.05 were indicated as *p < 0.05, **p < 0.01, ***p < 0.001.

## Additional Information

**How to cite this article**: Lee, A. S. *et al*. Effects of Prolonged GRP78 Haploinsufficiency on Organ Homeostasis, Behavior, Cancer and Chemotoxic Resistance in Aged Mice. *Sci. Rep.* **7**, 40919; doi: 10.1038/srep40919 (2017).

**Publisher's note:** Springer Nature remains neutral with regard to jurisdictional claims in published maps and institutional affiliations.

## Supplementary Material

Supplementary Information

## Figures and Tables

**Table 1:** Numbers of Mice with Tumors.

Mouse Strain	Pure C57BL/6		Pure 129sv		Mixed C57BL/6*129sv	
Tumor Type	Wild-type Mice Grp78^+/+^	Heterozygous Mice Grp78^+/−^	Wild-type Mice Grp78^+/+^	Heterozygous Mice Grp78^+/−^	Wild-type Mice Grp78^+/+^	Heterozygous Mice Grp78^+/−^
Liver Adenoma/Carcinoma	2/19 (11%)	1/13 (8%)	0	3/9 (33%)	0	0
Lung Carcinoma	0	1/13 (8%)	1/10 (10%)	1/9 (11%)	1/5 (20%)	1/6 (17%)
Lymphoma	4/19 (21%)	0	0	0	1/5 (20%)	0
Soft Tissue (Skin)	0	0	1/10 (10%)	0	0	0
**Total Number of Mice with Tumors**	**6/19 (32**%)	**2/13 (15**%)	**2/10 (20**%)	**4/9 (44**%)	**2/5 (40**%)	**1/6 (17**%)

The number in the numerator is the number of mice that developed a tumor at that site; the number in the denominator is the total number of mice studied. None of the differences between the wild-type and heterozygous mice were nominally statistically significant (*p* > 0.05) based on Fisher’s Exact Test.

**Table 2:** Numbers of Mice with Inflammation.

Mouse Strain	Pure C57BL/6		Pure 129sv		Mixed C57BL/6*129sv	
Site with Inflammation	Wild-type Mice Grp78^+/+^	Heterozygous Mice Grp78^+/−^	Wild-type Mice Grp78^+/+^	Heterozygous Mice Grp78^+/−^	Wild-type Mice Grp78^+/+^	Heterozygous Mice Grp78^+/−^
No Inflammation	10/19 (53%)	2/13 (15%)	1/10 (10%)	3/9 (33%)	3/5 (60%)	4/6 (67%)
Liver Only	5/19 (26%)	4/13 (31%)	2/10 (20%)	1/9 (11%)	0	0
Lung Only	0	0	2/10 (20%)	0	2/5 (40%)	2/6 (33%)
Kidney Only	0	4/13 (31%)	0	2/9 (22%)	0	0
Other Single Organ	2/19 (11%)	1/13 (8%)	1/10 (10%)	0	0	0
Multiple Locations	2/19 (11%)	2/13 (15%)	4/10 (40%)	3/9 (33%)	0	0
**Total with Inflammation**	**9/19 (47**%)	**11/13 (85**%)	**9/10 (90**%)	**6/9 (67**%)	**2/5 (40**%)	**2/6 (33%)**

The number in the numerator is the number of mice that developed inflammation at the indicated sites; the number in the denominator is the total number of mice studied. None of the differences between the wild-type and heterozygous mice were nominally statistically significant (*p* > 0.05) based on Fisher’s Exact Test, except for the pure C57BL/6 kidney comparison (p = 0.020) which is not statistically significant after adjusting for a 0.05 false discovery rate.
