# Supplementary material for: Effects of Prolonged GRP78 Haploinsufficiency on Organ Homeostasis, Behavior, Cancer and Chemotoxic Resistance in Aged Mice
Source: Sci Rep. 2017 Feb 1;7:40919. doi: 10.1038/srep40919 (PMC5286507; doi:10.1038/srep40919)
Supplement: Supplementary Information [file srep40919-s1.docx]

**Supplementary Information**

**Effects of Prolonged GRP78 Haploinsufficiency on Organ Homeostasis, Behavior, Cancer and Chemotoxic Resistance in Aged Mice**

Amy S. Lee, Sebastian Brandhorst, Daisy F. Rangel, Gerardo Navarrete, Pinchas Cohen, Valter D. Longo, Jeannie Chen, Susan Groshen, Todd E. Morgan & Louis Dubeau

**Supplemental Table 1: Complete Blood Count Measurements of Mice of the Indicated Genotype, Gender and Genetic Background**

**C57BL/6 129sv**

_________________________________________________________ ________________________

**Complete**

**Blood Count +/+ male +/- male +/+ female +/- female +/+ male +/- male**

WBC (10^9^/L) 7.96 ± 9.38 7.53 ± 5.34 4.88 ± 2.93 14.60 ± 19.58 1.60 ± 0.85 7.80 ± 12.50

Lymph (10^9^/L) 5.01 ± 5.94 4.06 ± 3.45 2.72 ± 2.74 9.83 ± 17.26 0.35 ± 0.07 1.40 ± 1.18

Mon (10^9^/L) 0.33 ± 0.44 0.32 ± 0.30 0.16 ± 0.09 0.56 ± 0.78 0.10 ± 0.00 0.25 ± 0.37

Gran (10^9^/L) 2.61 ± 3.07 3.15 ± 2.16 2.00 ± 0.68 4.21 ± 2.69 1.15 ± 0.78 6.15 ± 11.12

Lymph (%) 62.79 ± 6.70 53.53 ± 17.36 48.98 ± 21.36 51.34 ± 23.77 25.80 ± 7.92 31.85 ± 21.26

Mon (%) 7.40 ± 9.35 4.69 ± 2.56 4.08 ± 1.66 4.07 ± 0.83 6.35 ± 2.90 3.90 ± 0.89

Gran (%) 33.01 ± 5.96 41.78 ± 16.63 46.94 ± 19.75 44.59 ± 23.23 67.85 ± 10.82 64.25 ± 21.09

RBC (10^12^/L) 5.52 ± 1.97 5.64 ± 1.77 6.62 ± 1.99 7.23 ± 1.09 5.75 ± 0.35 5.12 ± 1.08

HGB (g/L) 8.14 ± 2.85 8.50 ± 2.39 10.36 ± 2.41 11.21 ± 1.36 10.00 ± 0.42 8.05 ± 2.26

HCT (%) 24.01 ± 9.21 25.59 ± 8.38 31.24 ± 8.09 35.13 ± 4.98 34.60 ± 1.70 27.27 ± 8.41

MCV (fL) 43.19 ± 2.45 45.08 ± 3.26 48.24 ± 6.21 49.06 ± 5.50 60.30 ± 0.85 52.88 ± 7.25

MCH (pg) 14.74 ± 0.69 15.16 ± 0.99 15.98 ± 1.66 15.59 ± 1.21 17.35 ± 0.35 15.62 ± 1.73

MCHC (g/L) 34.36 ± 1.99 33.87 ± 2.76 33.30 ± 1.57 32.04 ± 2.56 28.85 ± 0.21 29.67 ± 0.97

RDW (%) 13.43 ± 1.43 13.90 ± 1.45 13.38 ± 0.95 14.10 ± 1.31 14.30 ± 0.00 15.62 ± 1.90

PLT (10^9^/L) 720.33 ± 641.24 1390.80 ± 1021.10 797.80 ± 213.92 715.00 ± 442.79 68.50 ± 2.12 111.70 ± 79.88

MPV (fL) 5.66 ± 0.53 5.82 ± 0.53 5.66 ± 0.48 5.90 ± 0.72 9.45 ± 0.21 7.22 ± 1.56

PDW 14.98 ± 0.48 15.06 ± 0.42 15.04 ± 0.67 15.19 ± 0.51 16.90 ± 0.57 15.92 ± 0.94

PCT (%) 0.26 ± 0.20 0.20 ± 0.18 0.44 ± 0.11 0.41 ± 0.22 0.10 ± 0.00 0.07 ± 0.05

Blood was collected from tail vein and whole blood parameters were evaluated. All data mean + SD.
